# Phylogeny and Functions of LOB Domain Proteins in Plants

**DOI:** 10.3390/ijms21072278

**Published:** 2020-03-26

**Authors:** Yuwen Zhang, Ziwen Li, Biao Ma, Quancan Hou, Xiangyuan Wan

**Affiliations:** 1 Zhongzhi International Institute of Agricultural Biosciences, Biology and Agriculture Research Center, University of Science and Technology Beijing, Beijing 100024, China; zhangyuwenqian@163.com (Y.Z.); liziwen@ustb.edu.cn (Z.L.); mabiao@ustb.edu.cn (B.M.); houquancan@ustb.edu.cn (Q.H.); 2 Beijing Engineering Laboratory of Main Crop Bio-Tech Breeding, Beijing International Science and Technology Cooperation Base of Bio-Tech Breeding, Beijing Solidwill Sci-Tech Co., Ltd., Beijing 100192, China

**Keywords:** LOB domain, *LBD* gene family, transcription factor, phylogenetic analysis, functional diversity, plant growth and development

## Abstract

Lateral organ boundaries (LOB) domain (*LBD*) genes, a gene family encoding plant-specific transcription factors, play important roles in plant growth and development. At present, though there have been a number of genome-wide analyses on *LBD* gene families and functional studies on individual LBD proteins, the diverse functions of *LBD* family members still confuse researchers and an effective strategy is required to summarize their functional diversity. To further integrate and improve our understanding of the phylogenetic classification, functional characteristics and regulatory mechanisms of LBD proteins, we review and discuss the functional characteristics of LBD proteins according to their classifications under a phylogenetic framework. It is proved that this strategy is effective in the anatomy of diverse functions of *LBD* family members. Additionally, by phylogenetic analysis, one monocot-specific and one eudicot-specific subclade of LBD proteins were found and their biological significance in monocot and eudicot development were also discussed separately. The review will help us better understand the functional diversity of LBD proteins and facilitate further studies on this plant-specific transcription factor family.

## 1. The Confusion in Understanding Functional Diversity of LBD Proteins

Proteins containing conserved Lateral Organ Boundaries (LOB) domain(s) are defined as LOB-domain (LBD) proteins. They are plant-specific transcription factors, existing in plant genomes from green algae to angiosperms. LBD proteins are comprised of a relatively conserved N-terminal region and a variable C-terminal region [1]. The N-terminal region includes an LOB domain that comprises a zinc finger-like motif (CX2CX6CX3C) for DNA-binding activity, a GAS block (Gly-Ala-Ser) and a leucine-zipper-like coiled-coil motif (LX6LX3LX6L) responsible for protein dimerization [2,3]. A conserved proline residue in the GAS block was revealed to play a critical role in the biological function of LBD proteins in *Arabidopsis* [4]. C-terminal region confers transcriptional activation/repression of target gene expression [5]. According to sequence similarities and phylogenetic analyses, LBD family members were classified into two subfamilies (Class I and Class II) [6,7]. Class I LBD proteins containing zinc finger-like motifs, GAS blocks and leucine-zipper-like coiled-coil motifs can be grouped into four clades (IA, IB, IC and IE), whereas Class II LBD proteins lacking intact leucine-zipper-like domain are divided into two clades (IIA and IIB) [6,7].

LBD proteins were originally thought to play key roles in lateral organ development in plants (systematically reviewed in [3], and partly summarized for root development in [8,9,10,11] and leaf development in [12]), based on which the gene names were determined. However, subsequent progresses in functional studies proved that LBD proteins not only serve as essential transcription factors to regulate plant organ development, but also play important roles in versatile functions during plant growth and development (systematically reviewed in [13] and partly summarized for plant defense in [14]), such as photomorphogenesis [15], pulvinus identity and petiole development [16,17,18], pathogen resistance [19,20,21], hormone response [9,22,23], metabolism regulation [24,25], and heading date [26]. Though the functional significance of LBD proteins has been well summarized in several reviews according to the biological roles in plant development and defense response processes (e.g., root development, leaf development, and plant defense), such diverse functions are still confusing and make it difficult to grasp the regulatory role of this plant-specific gene family encoding transcription factors. One possible reason for the above confusion is that novel functions of LBD proteins have been continuously reported in recent years, while the most fundamental reason is that a method used to effectively sort out function characteristics of LBD proteins is still lacking. By combining the results in functional studies and evolutionary researches of LBD family members, we found that *LBD* genes have similar molecular functions tending to be in the same phylogenetic clade. It indicates that summarization of *LBD* gene functions under a phylogenetic framework could effectively resolve the confusion on understanding the diverse functions of LBD proteins. Therefore, to get a comprehensive understanding of such a relatively large family, reviewing functional studies based on deep phylogenetic analysis from more genome-available species is necessary.

Here, we first summarized the classification and evolution of *LBD* gene family members based on previous reports and generated a newly identified *LBD* gene family dataset, reconstructing LBD phylogeny from 18 representative genome-available plant species, including four species with evolutionary significance (one kind of green algae, one kind of moss, one kind of fern and one kind of basal angiosperm) and 13 kinds of food and industrial crops (six monocots and seven eudicots), as well as *Arabidopsis thaliana* (Table 1). We found one monocot-specific and one eudicot-specific LBD protein subclade that were not reported previously. Then, based on functional studies on *Arabidopsis* LBD proteins and the phylogenetic relationships, conserved and diverse functions of *Arabidopsis* LBD proteins in each clade were recapitulated. Finally, we summarize and discuss the functions for almost all of the function-reported *LBD* genes so far (50 genes) according to their phylogenetic classifications. This review closes the gap between functional studies and evolutionary researches on the LBD family. More importantly, through the phylogenetic analyses and clustering analysis, functions of a large number of genes can be predicted to facilitate further research on plant LBD proteins.

## 2. The Phylogeny of LBD Family Proteins

**Figure 1:**
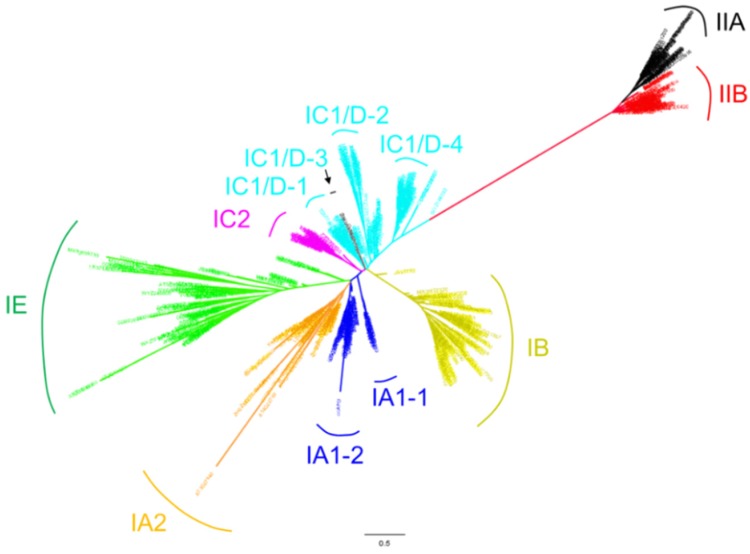
The phylogeny of LBD proteins in 18 genome-available plant species. The phylogenetic tree was reconstructed using aligned amino acid sequences of LOB domains. We reconstructed the hidden Markov model (HMM) of LBD domain using hmmbuild (HMMer package) with 163 seed sequences belonging to the *LBD* gene family (PF03195) in the Pfam database. All collected protein sequences of LBD candidates were aligned to the above HMM LBD domain by hmmalign (HMMer package). A maximum likelihood (ML) tree was constructed using the PhyML program with JTT model and 100 times of bootstrap replicates. Colored taxon names indicate the presence of Class IA1 (IA1-1, IA1-2) (blue), Class IA2 (orange), Class IB (yellow), Class IC1/D (IC1/D-1, IC1/D-2, and IC1/D-4)(cyan) without IC1/D-3 (black), Class IC2 (purple), Class IE (green), Class IIA (black) and Class IIB (red).

Genome-wide identification and evolutionary analysis of *LBD* gene families have been studied in more than a dozen plants [2,27,28,29,30,31,32,33,34,35,36,37]. Given that the genomes of 300 plant species were sequenced (plabiPD database), there remain a considerable number of genome-available species whose *LBD* genes deserve to be studied at genome level. In order to explore the functions of LBD proteins in more species, here a sophisticated method was used to identify the *LBD* genes in 18 genome-available species. Eight representative protein sequences from *Arabidopsis* and maize were used as queries to perform BLASTP searches against genome-wide predicted protein sequences of 18 genome-available plant species spanning from charophyta to angiosperms (Table 1). All candidate LBD protein sequences were examined by domain analysis using Pfam (http://pfam.xfam.org/) [38] with the default cut-off parameters. Only proteins with matched sequences covering at least 70% length of the complete LOB domain were regarded as LBD proteins. All the identified 823 LBD protein sequences used to reconstructed the phylogenetic tree shown in Figure 1 are provided in Supplementary Additional file 1.

Previous phylogenetic analyses of *LBD* genes indicate that they constitute a green plant-specific gene family that may have originated during the early evolution of charophyte algae [7]. Using the identified 823 LBD proteins from 18 plant species and nine well-studied LBD proteins from other species, we reconstructed a phylogenetic tree. Consistent with previously published topology results [6,7,39], LBD proteins can be classified into two major classes, Class I and Class II. Genes in Class I can be further grouped into four clades, Class IA (IA1-1, IA1-2, IA2), Class IB, Class IC (IC1/D-1, IC1/D-2, IC1/D-3 and IC1/D-4, IC2 ) and Class IE, and Class II consists of two clades (IIA and IIB) (Figure 1). This classification also works in LBD proteins from an individual plant (e.g., *Arabidopsis*).

A previous study had proved that the ancestral LBD proteins of the four clades of Class I and the subfamily Class IIB already exist in the last common ancestor of charophyte algae and land plants [7]. Here, we found LBD proteins from *Cylindrocystis brebissonii*, which belongs to charophyte algae located basally on the phylogenetic tree (see Figure 1 and some details in Part 4). The *C. brebissonii* LBD proteins distribute in clades of Class IA, Class IC and subfamily Class II, and each of them has two members. Phylogenetic results here show that *C. brebissonii* does not have Class IB type of LBD proteins, being consistent with some previous studies that proved that Class IB LBD proteins predominantly function in regulating lateral root development [40,41,42,43,44]. However, one previous comprehensive study demonstrated that *LBD* genes of charophyte algae are placed in all four clades of the Class I and Class II gene lineages [7], while this conclusion has not been proved by other studies. This may be due to the possibility that some other species belonging to charophyte algae possess Class IB and Class IE clades, and it is also possible that inaccurate sequence usage affected the analysis result. Our results also proved that both *Physcomitrella patens* and *Selaginella moellendorffii* have five major branches of *LBD* genes (Class II and four clades in Class I), which is consistent with the previous study [6]. Accordingly, maybe the initial radiation of the *LBD* gene family of five major branches was already established before the plants gained real roots. *Amborella trichopoda* is a known angiosperm that was the earliest one separated from other angiosperms. Its LBD proteins mainly distribute in the Class IA (IA1-2, IA2), Class IB, Class IC (IC1/D-2, IC1/D-4, IC1/D-5, and IC2), Class IE and Class II (IIA, IIB).

In our phylogenetic analyses, LBD proteins of the monocots and eudicots can separated from each other. The LBD proteins of *Arabidopsis* and maize are distributed in all major clades. However, at subclade level, the *Arabidopsis* and other eudicots have no protein distribution in the Class IC1/D-3 subclade (Figure 1 and some details in Part 4). Here, this subclade has eight homologs. Both wheat *(TraesCS4A02G297500, TraesCS4D02G014600)* and maize *(Zm00001d013625, Zm00001d033466)* have two members in this subclade, while rice *(BGIOSGA013249)*, barley *(HORVU4Hr1G002480)*, *Setaria italica (SETIT_037583mg) and* sorghum *(SORBI_3001G147000)* each has one. It indicates that the subclade may be monocot specific and these genes may play important roles in monocot plant development. However, no gene from this subclade has been cloned and investigated so far. Through searching the online databases of MaizeGDB (https://archive.maizegdb.org/) and BAR (http://bar.utoronto.ca/), we found that *Zm00001d013625, Zm00001d033466* and *BGIOSGA013249* are all highly expressed in shoot apical meristem (SAM). *Zm00001d013625* and *Zm00001d033466* are highly expressed in V5 stage (five leaves fully emerged) with similar expression patterns. Since the V5 stage is the time point when the stem tip growth point begins to develop the tassel primordia in maize [45], the monocot-specific genes are probably involved in inflorescence architecture development. In contrast, the Class IA1-1 is a eudicot-specific subclade (Figure 1 and some details in Part 4). Among the 14 identified Class IA1-1 genes, four belong to *G. max (GLYMA_18G025600, GLYMA_11G231500, GLYMA_14G057600* and *GLYMA_02G264500)*, two belong to each of *M. truncatula (MTR_3g071420* and *MTR_5g083010)* and *G. raimondii (B456_011G291500* and *B456_013G016900)*, one belongs to each of *A. thaliana (AT3G11090), B. vulgaris (BVRB_2g023440), B. napus (BnaC05g42060D), D. carota (DCAR_011832)*, *S. tuberosum (PGSC0003DMG400027718)* and *LjLOB1* from *Lotus japonicus*. *LjLOB1* was identified with strong expression at the bases of leaflet primordia, suggesting a potential function in leaf development of eudicots [46]. Taken together, both monocots and eudicots have their specific subclades. It may be generated from recent gene duplication events that occurred after dicot and monocot separation or result from gene loss events in the other evolutionary clade when dicots and monocots are separated.

## 3. Functional Conservation and Diversity of LBD Proteins in *Arabidopsis*

*Arabidopsis*, as a model plant, has the most in-depth studies on the functions of *LBD* genes. In *Arabidopsis*, the *LBD* gene family comprises 43 members, and at least 25 of them have been cloned and functionally characterized [3,13,47,48,49]. Here, we summarize the recent progress on the identification and characterization of LBD proteins in *Arabidopsis* and, combining the reported *LBD* gene functions with the phylogenetic results of *Arabidopsis LBD* family members, further investigate the substantial relationship between phylogenetic homology and functional similarity of *LBD* family genes.

**Figure 2:**
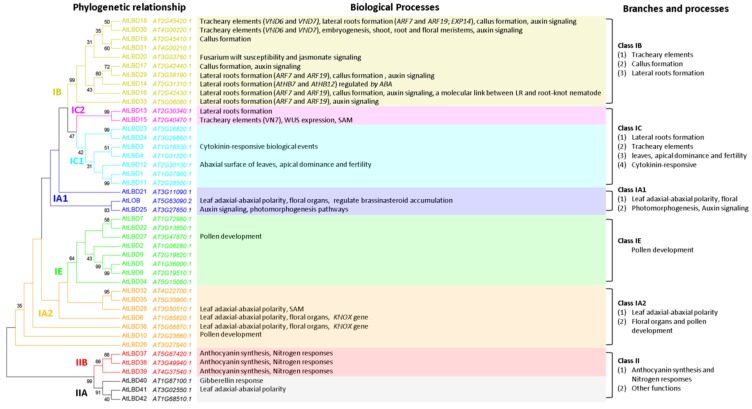
Phylogeny and functions of *Arabidopsis* LBD proteins. The phylogenetic tree was reconstructed in MEGA6 using aligned protein sequences of LOB domain regions in *Arabidopsis* by ML method with JTT model and Nearest-Neighbor-Interchange (NNI) heuristic searches based on an NJ (neighbour-joining) initial tree. Support values (>30) are shown next to the nodes. The colors of branches and gene names correspond to those in Figure 1.

By combining the function reports and evolutionary relationship of *Arabidopsis* LBD proteins, we found that *LBD* genes in the same phylogenetic clade tend to have similar molecular functions (Figure 2). Class IA *Arabidopsis* LBD proteins have main functions in aboveground organs (leaf adaxial–abaxial polarity and plant reproduction). Class IB LBD proteins play primary roles in the development of underground organs (lateral root formation). Class II LBD proteins mainly regulate anthocyanin synthesis and nitrogen responses. Molecular roles of Class IC and Class IE LBD proteins cannot be simply summarized due to the limited functional reports. Therefore, at least three of the five evolutionary branches of LBD proteins can be summarized in molecular functions under the phylogenetic framework. It is an effective strategy to resolve the confusion in understanding the functional diversity of *LBD* family members and can be used to reviewing the regulatory roles of LBD proteins.

## 4. Function Summarization Under Phylogenetic Framework

*LBD* genes were identified from many genome-available plant species, including *Arabidopsis* [2], rice [36], poplar [37], tomato [27], *Malus domestica* (apple) [28], *Medicago truncatula* [29], maize [30], grape [31], mulberry [32], barley [33], *Camellia sinensis* [34] and *Eucalyptus grandis* [35], with gene family sizes ranging from 24 to 58 genes. Recently, several studies focused on LBD protein identification and their novel roles are reported [26,50,51,52,53]. However, a large number of LBD proteins are still unexplored. So far, at least 50 *LBD* genes have been characterized to be involved in various processes of plant development and metabolism. A brief summary of these reported *LBD* genes is in Table 2. *Arabidopsis* was a model for the most in-depth studies on the function of the *LBD* gene family (25 *LBD* genes in Table 2). Besides, functions of some LBD proteins from other plant species were also investigated, including 10 in rice [3,13,26,53,54,55], four in maize [5,41,56,57], three in *L. japonicus* [16,46], two each in *M.truncatula* [16,58] and *E. grandis* [35], one in each of the five species (wheat [1], *H. vulgare* [50], citrus [20], pea [16] and apple [28]) (Table 2). In the next section, we summarize and discuss the major reports on the identification and characterization of LBD proteins in *Arabidopsis* and other plant species according to the reconstructed phylogenetic relationships.

### 4.1. Functions of LBD Proteins in Class IA Clade

**Figure 3:**
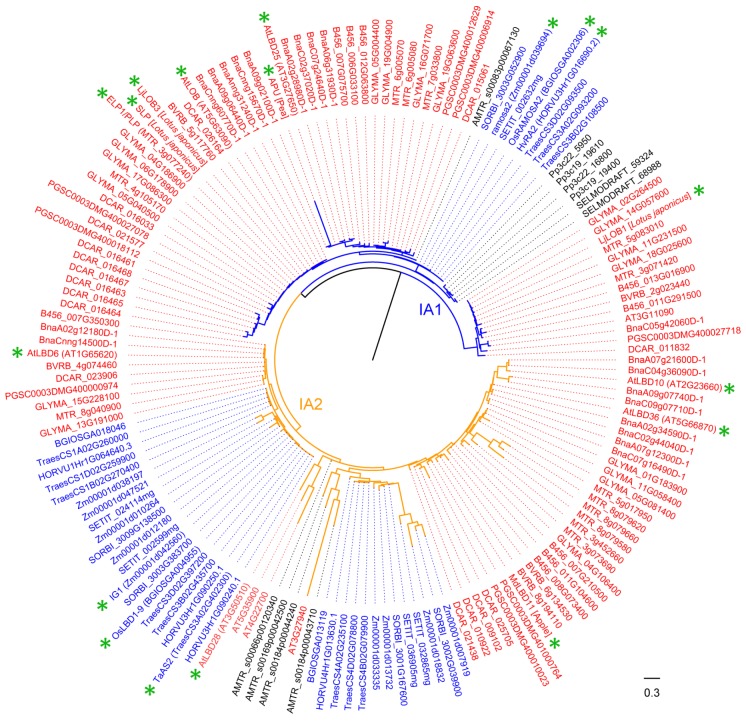
Phylogeny of LBD proteins from Class IA in 18 species. This phylogeny was reconstructed using Class IA LBD proteins identified in Figure 1 and according to the same method described in Figure 1 legend. “*” means functionally characterized LBD protein in Table 2 (Gene ID is in brackets for LBD proteins of 18 species, species name is in square brackets for LBD proteins of other species). LBD proteins of eudicots, monocots, and other plant species are marked in red, blue and black, respectively. Branch color corresponds to that in Figure 1.

In Class IA1-1 subclade, we identified only one characterized member, LjLOB1, from *L. japonicus* (Table 2). In Class IA1-2 subclade, there are 10 LBD proteins in *Arabidopsis*, and six of them were functionally characterized (AtLOB, AtLBD25/AtDDA1, AtLBD6/AS2, AtLBD36/AS1, AtLBD10 and AtLBD28) ( Figure 2 and Figure 3, Table 2).

AtLOB is one of the earliest identified members (founding members) of LBD proteins in *A. thaliana*. *AtLOB* loss-of-function mutant exhibits organ fusions under standard growth conditions [60]. Ectopic expression of *AtLOB* altered the size and shape of leaves, adaxial–abaxial polarity, and caused male and female sterility due to abnormal floral organs, suggesting its potential role in lateral organ development [2]. AtLOB can directly target the promoter region of *PHYBACTIVATION TAGGED SUPPRESSOR1* (*BAS1*) to negatively regulate brassinosteroid accumulation, resulting in limited growth in organ boundaries [59,60]. A basic helix-loop-helix (bHLH) member bHLH048 can interact with AtLOB and the interaction results in reduced affinity of AtLOB for the consensus DNA motif, which suggest that bHLH048 post-translationally regulates the function of AtLOB at lateral organ boundaries [61]. Another Class IA1-2 protein AtDDA1 is reported to play a role in photomorphogenesis through regulating light/dark-dependent hypocotyl elongation [15]. *AtDDA1* is expressed primarily in vascular tissues and its abundance is repressed by auxin and darkness treatments [15]. The *dda1-1* mutant, a conditional gain-of-function and semi-dominant allele, had a diminished auxin response and displayed aberrant hypocotyl elongation in the dark [15].

Three orthologous genes of *AtLOB* were identified in monocots, including *ramosa2*/*ra2* in maize, *HvRA2* in barley and *OsRA2* in rice (Table 2). The maize *ra2* is expressed at the axillary meristem initiation sites of the inflorescences and is involved in floral development [56,89]. In barley, *HvRA2* is a central player in establishing the inflorescence architecture of spikes, as well as in determining yield potential and grain number [50]. In rice, *OsRA2* was identified to regulate seed morphology and pedicel development in the panicle [54]. Moreover, *OsRA2* acts downstream of *RCN2* in regulating pedicel and branch lengths, but upstream of *RCN2* for control of the number of secondary branches. This indicates that branch number and length in the panicle are separately regulated by *OsRA2* through parallel pathways [54]. Additionally, sequence alignment of *RA2-like* with other LBD proteins reveals a grass-specific domain that is not found in *A. thaliana* in the C-terminus [56]. The expression pattern of *RA2* is conserved in rice, barley and maize, suggesting that RA2 may be a common factor critical for shaping the initial steps of grass inflorescence architecture [56]. Recently, crystal structural analysis of the LOB domain of wheat Ramosa2 reveals that RA2 shares some features different from other LBD proteins, and this study contributes to establish an atomic-scale mechanistic model for LBD proteins as transcriptional regulators in plants [1].

For eudicots, *ELP1/PLP* in *M. truncatula*, *APU* in pea (*Pisum sativum*), *SLP* and *LjLOB3* in *L. japonicus* also belong to Class IA1-2 (Table 2). Among them, ELP1, APU and SLP are orthologous proteins of AtLOB and were identified to regulate nastic leaf movement. Nastic leaf movement is generated by pulvinus and/or pulvinula, which are specialized motor organs located at the base of the petiole and petiolulae [90]. In *M. truncatula*, *ELP1/PLP* is specifically expressed in the pulvinus that gives rise to the motor organ [16]. The *ELP1/PLP* loss-of-function mutants in *M. truncatula* were impaired in pulvinus differentiation and failed to fold its leaflets in the dark [16,17]. Ectopic expression of *ELP1/PLP* resulted in dwarf plants with reduced petiole and rachises length, and the epidermal cells gained characteristics of motor organ epidermal cells [16]. The orthologs of *ELP1/PLP* in other legume species, including *APU* in pea and *SLP* in *L. japonicas*, confer similar regulation of pulvinulae development by a conserved molecular mechanism [16,17]. Furthermore, ELP1/PLP can be negatively regulated by *PHANTASTICA (MtPHAN*), as *MtPHAN* is required to maintain petiole identity by repressing the ectopic expression of *ELP1* [18]. In addition, LjLOB3 was strongly expressed at the bases of leaflet primordia, suggesting its potential function in leaf development in *L. japonicus* [46].

In *Arabidopsis*, AtLBD6/AS2, AtLBD36/AS1, AtLBD10 and AtLBD28 are classified into Class IA2 subclade (Figure 2, Table 2). *AS2*, the other founding member of *LBD* gene family, can form complexes with different proteins to regulate various aspects of plant growth and development [62,63,64,65]. AS2 physically associates with AS1 to form a repressor complex that regulates the polarity and morphologies of leaf, the inflorescence architecture and fertility, and the differentiation of shoot apical meristem [62,63,64,65]. AS2 interacts with AtLBD30/JLO to regulate the expressions of several *PIN-FORMED (PIN)* genes encoding Aux efflux facilitators [80,91,92]. AS2, AS1 and JLO can form a trimeric protein complex involved in organ boundary establishment via the negative regulation of *KNOX* gene expression [66]. In addition, AS2 alone can promote gibberellin (GA) synthesis via repression of *KNOX* gene [93]. AtLBD28 might affect the polarity and morphologies of leaf and the differentiation of shoot apical meristem [47]. However, AtLBD10 has a unique biological function different from the other three well-studied members. It is involved in microspore polarization prior to the first asymmetric division, as well as in germ cell mitosis [68,69]. The *lbd10* mutants had aborted pollen grains at a ratio of 12.7%, indicating that AtLBD10 is important for *Arabidopsis* pollen development [68].

In other plants, OsAS2 and OsIG1 in rice, IG1 in maize, TaAS2 in wheat and MdLBD11 in *M. domestica* are all classified into Class IA2 subclade (Table 2), and they are orthologous proteins of *Arabidopsis* AS2. OsIG1 can regulate shoot differentiation and leaf development [70], and the development of floral organs and megagametophyte in rice [71]. The maize IG1 was characterized as a key regulator of leaf adaxial–abaxial patterning, as well as embryo sac development. Furthermore, mutant of *ig1* leads to male sterility in some genetic backgrounds (A158, W23, W64A and W22) and variable male sterility in other genetic background (Mo17) [57]. Ectopic expression of wheat *TaAS2* in *Arabidopsis* leads to the adaxialization of abaxial mesophyll tissues and alterations of the vascular patterns in leaves and petioles [72]. The *M. domestica* MdLBD11 was highly similar to *Arabidopsis* AS1 and AS2 in molecular functions in regulating leaf and flower development. Overexpression of *MdLBD11* in *Arabidopsis* resulted in upward curling leaves, delayed flowering, downward pointing flowers and abnormal siliques and other phenotypic changes [28].

Taken together, 19 LBD proteins in Class IA clade were identified from nine plant species. Most of the Class IA members function in aboveground organ development, including the differentiation of SAM, the size, shape and adaxial–abaxial polarity of leaves, inflorescence architecture, pollen development, pulvinus development, nastic leaf movement, and photomorphogenesis process. Though some genes were identified in other plants, the functions of their homologous genes in our selected six monocots and eight eudicots are still unclear. In order to get a clear phylogenetic relationship between well-studied and function-unknown genes, a more detailed phylogenetic tree for Class IA clade is shown (Figure 3), based on which functions of uninvestigated genes belonging to listed species in the phylogeny can be predicted by taking their well-studied homologs as references.

### 4.2. Functions of LBD Proteins in Class IB Clade

In *Arabidopsis*, 10 LBD proteins are classified into Class IB clade, nine of which have been characterized (Figure 2, Table 2). Their functions are mainly involved in root development, callus formation and differentiation of tracheary elements, and the resistances to *Fusarium oxysporum* and root-knot nematode (RKN) pathogenesis.

*LBD* genes play important roles in regulating root development. They regulate lateral root (LR) formation in *Arabidopsis* [43,49,73] and *Medicago* [58], crown root (CR) [42] and adventitious root (AR) [84] development in rice, and shoot-borne root initiation in maize [5,41,85]. In *Arabidopsis*, *AtLBD16*, *AtLBD18*, *AtLBD29* and *AtLBD33* genes act downstream of *ARF7*/*19*-mediated auxin signal transduction cascade to control the LR formation [40,44,75,93,94,95,96] (Figure 2, Table 2). *AtLBD29* was expressed in the LR primordia [40], *AtLBD16* was expressed throughout the young LR [44,97], and expression of *AtLBD18* was restricted to the base of the LR [44]. AtLBD18 forms a heterodimer with AtLBD33 to reactivate the auxin-dependent division [76]. It was shown that the auxin influx carriers AUXIN1 (AUX1) and LIKE-AUXIN3 (LAX3) are required for the auxin-induced expression of *AtLBD16* and *AtLBD18* to control LR development during various stages in *Arabidopsis* [98,99,100]. The ARF7/19-AtLBD16/18 transcriptional module was also identified as playing an important role in AR formation in *Arabidopsis* [101]. In *Arabidopsis*, initiation of LR formation starts from the founder cells’ asymmetric division, and through subsequent cell proliferation and differentiation to form new primordia. *AtLBD16* is a key member for LR formation in an auxin-dependent manner [11,102]. LR initiation requires the sequential induction of transcription factor gene *AtLBD16* and its target *PUCHI* [102]. AtLBD18 and ARFs form a double positive feedback loop, as AtLBD18 can not only bind to the ARF19 promoter directly but also interacts with ARF7 and ARF19 [101]. These feedback loops may contribute to the continued LR growth in response to auxin in *Arabidopsis* [101]. The coiled-coil motifs in AtLBD16 and AtLBD18 transcription factors determine their DNA-binding properties, including DNA-binding diversity, specificity and affinity, which functions in the transcriptional regulation of different cellular processes and biological pathways in *Arabidopsis* [103]. By contrast, *AtLBD14* was not responsive to auxin, but it was downregulated by ABA and participates in ABA-mediated regulation of LR formation [51,73] (Figure 2, Table 2).

Callus formation, the initial step of the typical in vitro plant regeneration triggered by auxin, shares a similar genetic pathway with LR development [104,105]. In *Arabidopsis*, the LBD proteins that control LR formation, such as AtLBD16, AtLBD17, AtLBD18, and AtLBD29 (Figure 2, Table 2), were identified as key regulators of callus induction in various organs, but the molecular mechanisms of auxin-induced callus formation remain largely elusive [74]. Recently, it was shown that *Arabidopsis* basic region/leucine-zipper-motif 59 (AtbZIP59) transcription factor forms complexes with LBD proteins to modulate auxin-induced callus formation [52]. Auxin can stabilize AtbZIP59 and enhance its interaction with AtLBD16, and AtbZIP59–LBD16 complex directly targets an *FAD-binding Berberine* (*FAD-BD*) gene’s promoter and regulates its transcription [52]. Furthermore, the WOX11-LBD16 pathway was proved to promote pluripotency acquisition in callus cells [106]. Using ChIP sequencing (ChIP-seq) and RNA sequencing (RNA-seq) approaches, more than 350 target genes of AtLBD29 were identified participating in the regulation of cell reprogramming during callus formation. AtLBD29 rapidly activates genes that are involved in reactive oxygen species (ROS) and lipid metabolism, methylation and cell wall hydrolysis, but suppresses most of the light-responsive genes [78]. Besides, a recent study suggests that AtLBD19 may play an important role in coordinating callus formation in *Arabidopsis* along with other AtLBD members [77]. Moreover, AtLBD16 and AtLBD29 are required for AR formation from wounded or detached plant tissues, a different auxin-driven process called “de novo root organogenesis” [105]. WOX11 acts redundantly with its homolog WOX12 and directly responds to a wounding-induced auxin that accumulated in and surrounding the procambium to activate *AtLBD16* and *AtLBD29* expressions, which lead to the first step in cell fate transition from a leaf procambium or its nearby parenchyma cell to a root founder cell [105]. 

JAGGED LATERAL ORGANS (JLO) is a dosage-dependent regulator of cell specification and organ patterning throughout plant development [66,79,91] (Table 2). Loss of *JLO* resulted in arrested seedling development at early stages and even embryo lethality [79,91], while compromised JLO activity leads to abnormal organ initiation and patterning of leaf and flower [66]. AS1, AS2, and JLO form a trimeric protein complex to involve in the formation of organ boundaries by negatively regulating *KNOX* expression [66]. JLO transcriptionally regulates several *PIN* genes (encoding auxin efflux facilitators) independently or together with AS2 [79,91,92]. JLO also coordinates root meristem identity through activating AP2 transcription factors (encoded by *PLT* genes) that further regulate *PIN* expression [91,107,108]. In addition, JLO is involved in hypophysis specification and primary root meristem formation during embryogenesis by influencing the action of the auxin-signaling module BDL/IAA12-MP/ARF5 [91]. The versatile functions of JLO in plant development are probably due to its involvement in regulating auxin distribution and signaling [13]. Besides, *JLO* and *AtLBD18* are recently duplicated genes involved in the differentiation of tracheary elements of xylem vessels via the positive feedback regulation of NAM/ATAF/CUC (NAC) proteins [76,109].

In addition to the functions in plant development, some Class IB clade members of *Arabidopsis* also play important roles in response to fungal pathogens and soil nematodes [14]. *AtLBD20*, a predominantly root-expressed *LBD* gene in *Arabidopsis*, is a negative regulator of both *F. oxysporum* resistance and a subset of jasmonic acid (JA) responses [19]. Loss-of-function mutants of *Atlbd20* display increased resistance to the root-infecting vascular wilt pathogen *F. oxysporum*, indicating that AtLBD20 acts as a negative regulator of JA-regulated pathogen defense. Besides, AtLBD16 is regulated by auxin in galls and induced by nematode secretions (including auxin compounds and can trigger changes in pericycle cells through inducing the expression of *AtLBD16*) [21]. AtLBD16 and its co-regulated genes integrate the auxin signaling cascades in both LR and callus formation, establishing the molecular links between lateral root development and RKN pathogenesis [110] (Figure 2, Table 2). Furthermore, beet necrotic yellow vein virus (BNYVV) can hijack some auxin-regulated pathways that are dependent on several LBD transcription factors to cause rhizomania of sugar beet [111]. Therefore, Class IB clade *LBD* genes are crucial molecular targets for plant pathogen invasion.

**Figure 4:**
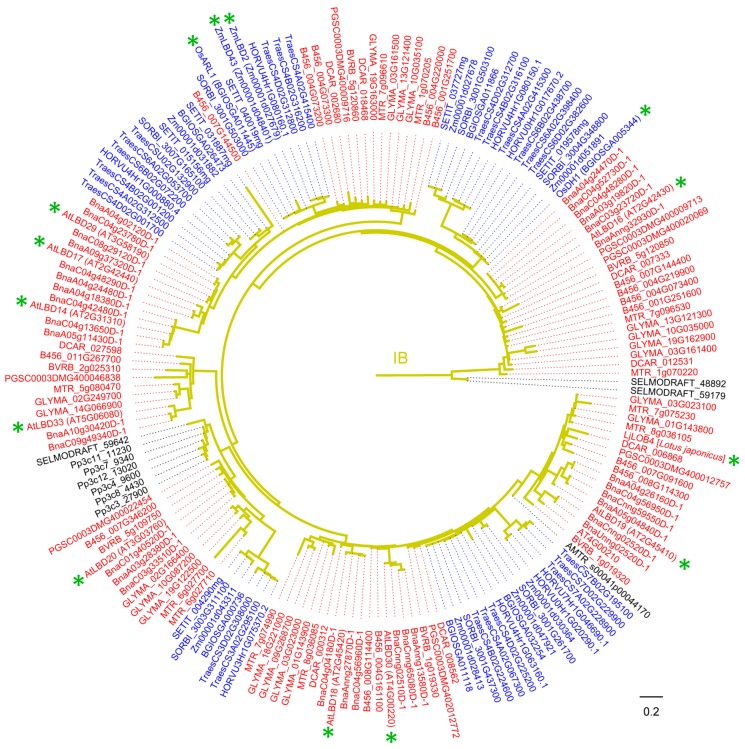
Phylogeny of LBD proteins from Class IB in 18 species. This phylogeny was reconstructed using Class IB LBD proteins identified in Figure 1 and according to the same method described in Figure 1 legend. “*” means functionally characterized LBD protein in Table 2 (Gene ID is in brackets for LBD proteins of 18 species, species name is in square brackets for LBD proteins of other species). LBD proteins of eudicots, monocots, and other plant species are marked in red, blue and black, respectively. Branch color corresponds to that in Figure 1.

For other plants, five *LBD* genes have been characterized (Table 2 and Figure 4). *LBD* genes *Crl1 (Arl1)* and *OsARL1* in rice are involved in the formation of monocot-specific CR and AR, respectively [42,82,83,84]. The *Crl1* orthologs in maize, *RTCS* and *RTCL,* are responsive to auxin and regulate in embryonic seminal and post-embryonic shoot-borne root initiation [5,41,85]. *OsDH1*, the first reported *LBD* gene with tissue-specific and temporal expression patterns, is involved in rice floral organ development [81]. In *L. japonicus*, *LjLOB4* was expressed at the boundaries between whorls in developing floral buds, suggesting a potential function during floral development [46].

Taken together, most of the Class IB clade members are involved in auxin-related biological processes, including root development, differentiation of tracheary elements, callus formation, root-related resistance to plant pathogen, and the development of leaves and flowers. A more detailed phylogenetic tree was reconstructed from Class IB LBD proteins in 18 plant species, facilitating the further research on the functions of unknown members (Figure 4).

**Figure 5:**
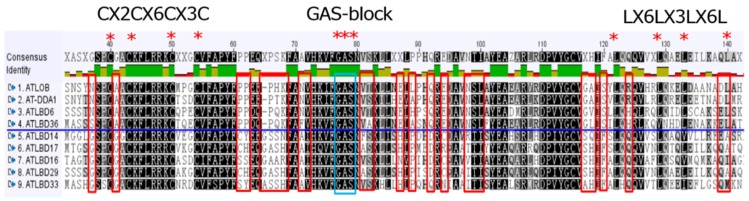
Sequence comparison of selected Class IA and Class IB LBD proteins. Red frame indicates conserved amino acid residue within each clade. “*” indicates conserved amino acid residues across different clades.

Interestingly, as mentioned above, Class IA LBD proteins mainly function in the regulation of aboveground organs; contrarily, a set of Class IB members regulate root development and root-related biological processes. In order to find the sequence difference potentially responsible for the functional diversities between Class IA and Class IB *LBD* genes, representative protein sequences from the two clades were compared in the LOB domains (Figure 5). At some positions (highlighted with red frames), amino acid residues are conserved within each of the two classes but are divergent between them. Amino acids at these positions may determine the specificity of target genes and explain functional differences between the two classes of *LBD* genes in regulating the aboveground and the below-ground organ development, respectively.

### 4.3. Functions of LBD Proteins in Class IC Clade

In *Arabidopsis*, four out of 11 Class IC LBD proteins (AtLBD3, AtLBD12, AtLBD13 and AtLBD15) were characterized (Figure 2, Table 2). AtLBD3 and AtLBD12 belong to subclade IC1/D, while AtLBD13 and AtLBD15 belong to subclade IC2. AtLBD3 is expressed at the base of shoot lateral organs and root, and it is temporally and spatially regulated by the plant hormone cytokinin in a manner dependent on the His-Asp phosphorelay signal transduction [23]. *AtLBD3* overexpression transgenic lines commonly display a dwarfism phenotype with stunted rosette leaves, inflorescences and flowers [23]. AtLBD12 is involved in the growth of abaxial leaf surface, apical dominance and fertility as identified by using an activation tagging line [86]. The expression of AtLBD13 is not responsive to auxin and ABA, but can be downregulated by brassinolide treatment, which indicates that AtLBD13 may have a unique role in lateral root formation [49]. AtLBD15 is involved in SAM development through regulating WUSCHEL (WUS) expression and affecting xylem establishment [48,87].

In rice, *LBD12-1* loss-of-function mutant had a larger SAM under salt stress, whereas overexpression of *LBD12-1* resulted in reduced SAM size through repressing *AGO10* expression [53]. *OsLBD3-7* is involved in the regulation of rice leaf rolling as its overexpression leads to narrow and adaxially rolled leaves [55]. In citrus, *CsLOB1*, the ortholog of *AtLBD1* and *AtLBD11*, is a general susceptibility gene for citrus bacterial canker (CBC) disease incited by multiple *Xanthomonas* species [20]. In *E. grandis*, two Class IC LBD proteins were identified. Overexpressing *EgLBD37* leads to the significant increase of secondary xylem, and overexpressing *EgLBD29* results in greatly increased phloem fiber production [35], which suggests that *E. grandis LBD* genes may play important roles in secondary growth (Table 2). 

**Figure 6:**
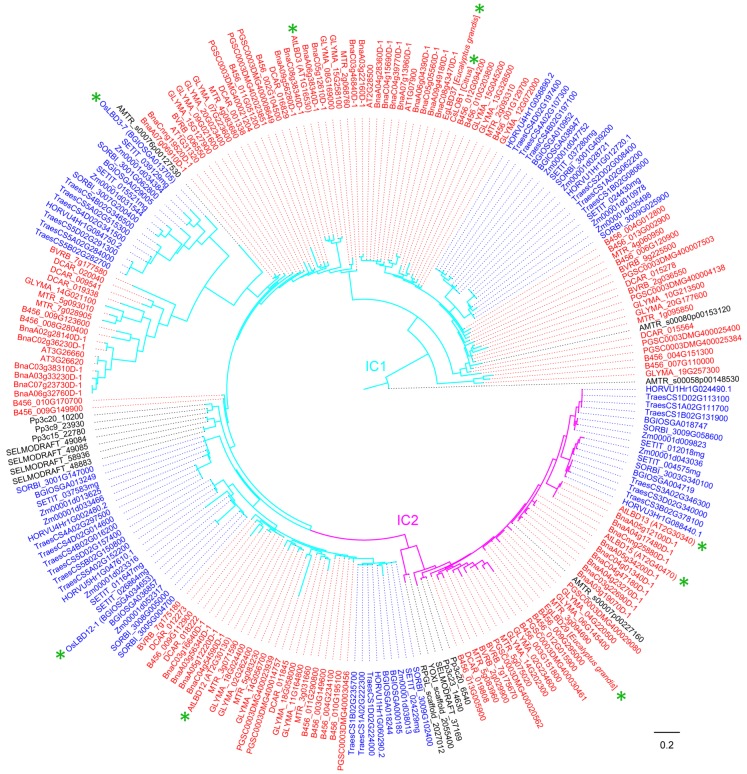
Phylogeny of LBD proteins from Class IC in 18 species. This phylogeny was reconstructed using Class IC LBD proteins identified in Figure 1 and according to the same method described in Figure 1 legend. “*” means functionally characterized LBD protein in Table 2 (Gene ID is in brackets for LBD proteins of 18 species, species name is in square brackets for LBD proteins of other species). LBD proteins of eudicots, monocots, and other plant species are marked in red, blue and black, respectively. Branch color corresponds to that in Figure 1.

Collectively, the Class IC clade LBD proteins mainly regulate cytokinin-mediated plant growth, abaxial surface of leaves, apical dominance, fertility, and secondary growth. A more detailed phylogenetic tree of Class IC LBD proteins was reconstructed for future researches (Figure 6).

### 4.4. Functions of LBD Proteins in Class IE Clade

**Figure 7:**
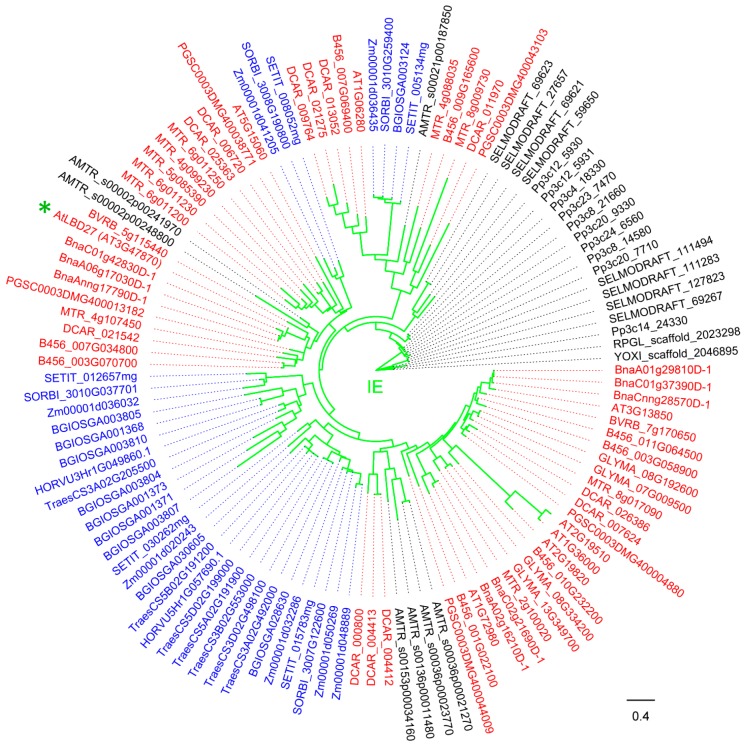
Phylogeny of LBD proteins from Class IE in 18 species. This phylogeny was reconstructed using Class IE LBD proteins identified in Figure 1 and according to the same method described in Figure 1 legend. “*” means functionally characterized LBD protein in Table 2 (Gene ID is in brackets for LBD proteins of 18 species, species name is in square brackets for LBD proteins of other species). LBD proteins of eudicots, monocots, and other plant species are marked in red, blue and black, respectively. Branch color corresponds to that in Figure 1.

Among Class IE LBD members, only *Arabidopsis* AtLBD27 was functionally studied (Figure 2, Table 2). AtLBD27 plays an essential role in pollen development [68,69,112]. Flowering plants have a complex life cycle that involves a switch between a multicellular (2n) sporophyte generation and a (n) gametophyte generation. AtLBD27 is required for correct initiation and orientation of the polarized microspore’s asymmetric division to generate bicellular pollen, as the *lbd27* mutant produces aberrant microspores with increased cell expansion, delayed mitosis entry and altered nuclear division orientation phenotypes. [112,113]. AtLBD10, which belongs to Class IA, can also affect the pollen development as mentioned above. The *lbd10* or *lbd27* single mutant had aborted pollen grains at a ratio of 12.7% and 70% respectively, whereas all pollens in the *lbd10 lbd27* double mutants were aborted, indicating that both AtLBD10 and AtLBD27 are essential for *Arabidopsis* pollen development [68]. Figure 7 shows a more detailed phylogenetic tree for Class IE clade LBD proteins.

### 4.5. Functions of LBD Proteins in Class II Clade

**Figure 8:**
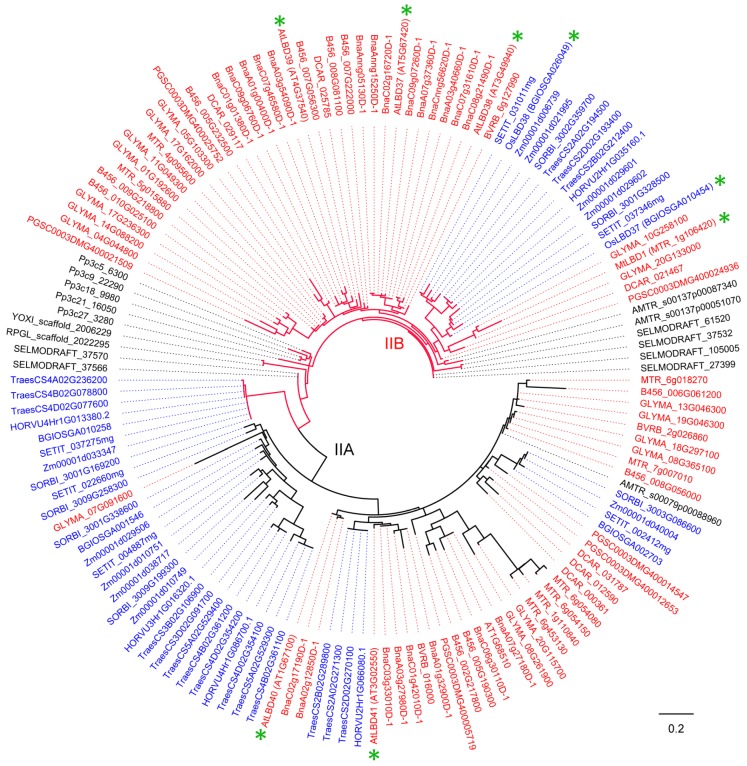
Phylogeny of LBD proteins from Class II in 18 species. This phylogeny was reconstructed using Class II LBD proteins identified in Figure 1 and according to the same method described in Figure 1 legend. “*” means functionally characterized LBD protein in Table 2 (Gene ID is in brackets for LBD proteins of 18 species, species name is in square brackets for LBD proteins of other species). LBD proteins of eudicots, monocots, and other plant species are marked in red, blue and black, respectively. Branch color corresponds to that in Figure 1.

There are six *Arabidopsis* LBD proteins in the Class II clade and they can be further divided into two subclades, Class IIA and Class IIB (Figure 2 and Figure 8, Table 2). In *Arabidopsis*, two of Class IIA LBD proteins (AtLBD40 and AtLBD41) were functionally studied. *AtLBD40* is reported to be downregulated by GA but upregulated by DELLA proteins [22]; however, no more detailed analysis of this gene is available at present. *AtLBD41* was detected in the adaxial and internal domain between the ab-adaxial domains of leaves, indicating it might play a role in the specialization of adaxial cells in *Arabidopsis* lateral organs [88]. Up to now, there is no more to report about Class IIA members in other plants.

For Class IIB, three members (AtLBD37, AtLBD38 and AtLBD39) were identified in *Arabidopsis*, with main functions in anthocyanin synthesis and nitrogen (N) metabolism (Figure 8). N and nitrate (NO_3_^-^) can regulate many aspects of plant metabolism, growth, and development. In *Arabidopsis*, N and NO_3_^-^ suppress anthocyanin synthesis via the induction of *AtLBD37*, *AtLBD38* and *AtLBD39* expressions [24,114]. The three *LBD* genes act as repressors of N availability signals by negatively regulating two anthocyanin synthesis regulators (*PAP1* and *PAP2*) and N-responsive genes [24]. Recent studies reported that AtLBD37 can interact with the development regulators of TPL/TPR proteins and miP1a/b/TPL complex to repress flowering by recruiting CONSTANS (CO, a potent regulator of flowering time) [115,116].

For other plants, three Class IIB LBD proteins were identified, including two from rice and one from *Medicago* (Figure 8, Table 2). In rice, the metabolomic and transcriptomic analysis on *OsLBD37* overexpressing plants revealed that OsLBD37 is also associated with nitrogen metabolism [25]. *OsLBD37* and *OsLBD38*, two homologs of *Arabidopsis AtLBD37*, serve as negative regulators of rice heading date [26]. Overexpression of *OsLBD37* and *OsLBD38* delayed heading date and increased yield via downregulating the florigen genes *Hd3a* and *RFT1* and the key regulator of heading date *Ehd1* [26]. In *Medicago*, homeobox 1 (HB1, belonging to the HD-Zip family) directly binds to a CAATAATTG *cis*-element present in the promoter of *LBD1* to regulate the LR emergence by auxin [58]. Figure 8 shows a more detailed phylogenetic tree for Class II clade LBD proteins.

## 5. Conclusions and Perspectives

LBD proteins, a family of plant-specific transcription factors, play important roles in controlling plant development and responding to external stimuli. In this review, we identified 823 LBD proteins from 18 high-quality plant genomes containing green algae (*C. brebissonii*), moss (*P. patens*), fern (*S. moellendorffii*), and angiosperms (*A. trichopoda*, eight eudicots and six monocots). The phylogenetic results are mainly consistent with previous studies. However, through detailed analyses of the proteins from different branches, we found the Class IC1/D-3 subclade is specific to monocots and Class IA1-1 subclade is specific to eudicots. All the eight LBD proteins in Class IC1/D-3 subclade are from monocots, and three genes *Zm00001d013625, Zm00001d033466* and *BGIOSGA013249* are all highly expressed at the time point when the stem tip growth point begins to develop the tassel primordia. Thus, the Class IC1/D-3 subclade may be involved in the biological process of inflorescence architecture development. The Class IA1-1 is specific for eudicots and only one gene in this clade has been functionally investigated with strong expression at the bases of leaflet primordia, indicating that the subclade may play important roles in eudicot leaf development. Therefore, it would be interesting to precisely identify the functions of LBD proteins in Class IC1/D-3 and Class IA1-1 to explore whether these proteins are key regulators in determining morphogenesis.

We summarize the characterized gene functions based on a phylogenetic framework, which effectively facilitates understanding of diverse functions of LBD family proteins. We found Class IA is mainly involved in regulating the development of aboveground organs (leaves, stems, flowers) and their related biological and abiotic reactions (inflorescence architecture and photomorphogenesis). Class IB is mainly involved in regulating the development of underground organs (lateral roots, crown roots and adventitious roots) and their related biological processes (root-related plant diseases and insect pest responses). The subclade Class IIB LBD proteins are mainly involved in nitrogen metabolism in *Arabidopsis* and rice. The number of well-studied genes in Class IC and Class IE is small and their molecular mechanism in transcriptional regulation is still unknown. At present, the majority of well-studied LBD proteins belong to *Arabidopsis*, and few LBD proteins are characterized in other plants. Therefore, one major task for further studies is to investigate *LBD* gene functions in other plants, and another one is to characterize individual LBD proteins by elucidating their regulatory mechanisms and involved pathways, such as disclosing *cis*-regulatory elements, identifying protein partners, and detecting downstream targets and upstream regulators.

## Figures and Tables

**Table 1:** Information on the 18 plant species for Lateral organ boundaries (LOB) domain (*LBD*) family analyses.

General Classification	Species	Gene Numbers in *LBD* Family	Data Source	Gene ID Prefix
Green alga	*Cylindrocystis brebissonii*	6	China National GeneBank Database	RPGL/YOXI
Moss	*Physcomitrella patens*	30	Gramene Database	Pp
Fern	*Selaginella moellendorffii*	24	Gramene Database	SELMO
Basal angiosperm	*Amborella trichopoda*	20	Gramene Database	AMTR
Eudicots	*Arabidopsis thaliana*	43	Gramene Database	AT
	*Beta vulgaris*	23	Gramene Database	BVRB
	*Brassica napus*	118	Gramene Database	Bna
	*Daucus carota*	54	Gramene Database	DCAR
	*Glycine max*	79	Gramene Database	GLYMA
	*Gossypium raimondii*	65	Gramene Database	B456
	*Medicago truncatula*	57	Gramene Database	MTR
	*Solanum tuberosum*	39	Gramene Database	PGSC
Monocots	*Hordeum vulgare*	28	Gramene Database	HORVU
	*Oryza sativa*	37	Gramene Database	BGIOSGA
	*Setaria italica*	32	Gramene Database	SETIT
	*Sorghum bicolor*	33	Gramene Database	SORBI
	*Triticum aestivum*	86	Gramene Database	TraesCS
	*Zea mays*	49	Gramene Database	Zm00001d

**Table 2:** Functionally characterized *LBD* genes and their phylogenetic classification. “-” means “no reports so far”.

Classes	Species	Genes	Biological Processes	Target or Downstream Genes	References
Class IA	*Arabidopsis*	*AtLBD25*/ *DDA1*	Auxin signaling, photomorphogenesis	- (no reports so far)	[15]
	*Arabidopsis*	*AtLOB*/ *ASL4*	Leaf adaxial–abaxial polarity, floral organ development, brassinasteroid accumulation	*BAS1*	[2,59,60,61]
	*Arabidopsis*	*AtLBD6*/*AS2*	Leaf adaxial–abaxial polarity, floral organ development	*BP*, *KNAT2*	[62,63,64,65,66]
	*Arabidopsis*	*AtLBD36*/*AS1*			
	*Arabidopsis*	*At* *LBD10*	Pollen development	-	[67,68,69]
	*Arabidopsis*	*At* *LBD28*	Leaf adaxial–abaxial polarity, SAM genesis	-	[47]
	Rice	*OsLBD1-9*/ *OsAS2;* *OsIG1*	Shoot differentiation, leaf development	-	[70]
			Empty-glume identity, floral organ number control, female gametophyte development	*EG1*, *OsMADS1* and *OsMADS6*	[71]
	Rice	*OsRAMOSA2*/ *OsRA2*	Panicle architecture establishment	*LAX1, RCN2*	[54]
	Maize	*ramosa2*/ *ra2*	Inflorescence architecture establishment	-	[56]
	Maize	*IG1*	Embryo sac, leaf and tassel development	*KN1, RS1, LG3, LG4A/B*, and *KNOX3*	[57]
	Wheat	*TaAS2*	Leaf adaxial–abaxial polarity	-	[72]
	Barley	*Vrs4* */* *HvRA2*	Control lateral spikelet fertility and row-type pathway	*Vrs1, HvSRA* and *T6P*	[50]
	Apple	*MdLBD11*	Leaf and flower development	-	[28]
	*Lotus japonicus*	*LjLOB1*	Compound leaf development, Floral development	-	[46]
		*LjLOB3*			
	*Lotus japonicus*	*SLP*	Pulvinus development, leaf movement	-	[16]
	*Medicago*	*ELP1/* *PLP*	Pulvinus development, leaf movement	-	[16,17,18]
	Pea	*APU*	Pulvinus development, leaf movement	-	[16]
Class IB	*Arabidopsis*	*AtLBD14*	ABA-mediated lateral roots formation	*AtHB7* and *AtHB12*	[51,73]
	*Arabidopsis*	*AtLBD16*	Lateral roots formation, callus formation, gall formation	*FAD-BD, ARF7* and *ARF19*	[21,40,43,44,52,74]
	*Arabidopsis*	*AtLBD17*	Auxin-induced callus formation	-	[52,74]
	*Arabidopsis*	*AtLBD18*	Tracheary element differentiation, lateral roots formation, callus formation, auxin signaling	*ARF7* and *ARF19, EXP14, E2Fa, VND6* and *VND7*	[44,74,75,76]
	*Arabidopsis*	*AtLBD19*	Callus formation	-	[77]
	*Arabidopsis*	*AtLBD20*	Fusarium wilt susceptibility, jasmonate signaling	*MYC2, Thi2.1*, *VSP2*, and *PDF1.2*	[19]
	*Arabidopsis*	*AtLBD29*	Lateral roots formation, callus formation, auxin signaling	*ARF7* and *ARF19*	[40,52,74,78]
	*Arabidopsis*	*AtLBD30*/ *JLO*	Tracheary element differentiation, embryogenesis, shoot, root and floral meristems development, auxin signaling	*VND6* and *VND7*	[66,76,79,80]
	*Arabidopsis*	*AtLBD33*	Auxin-mediated lateral roots formation	*ARF7* and *ARF19, E2Fa*	[75]
	Rice	*OsDH1*	Floral organ development	-	[81]
	Rice	*Crownrootless1* (*crl1*); *OsARL1*	Auxin-mediated crown root formation	*FSM, GTE4* and *MAP*	[42,82,83]
			Auxin-mediated adventitious root formation	-	[84]
	Maize	*ZmLBD2*/ *RTCS*	Embryonic seminal and post-embryonic shoot-borne root initiation, auxin signaling	*ZmArf34*	[5,41,85]
	Maize	*ZmLBD43*/ *RTCL*			
	*Lotus japonicus*	*LjLOB* *4*	Compound leaf development, Floral development	-	[46]
Class IC	*Arabidopsis*	*AtLBD3*/ *ASL9*	Cytokinin-responsive biological events	-	[23]
	*Arabidopsis*	*At* *LBD12*	Growth of abaxial leaf surface, apical dominance and fertility	-	[86]
	*Arabidopsis*	*At* *LBD13*	Lateral root formation	-	[49]
	*Arabidopsis*	*At* *LBD15*	Tracheary element development, SAM development	*WUS*	[48,87]
	Rice	*OsLBD3-7*	Leaf rolling	-	[55]
	Rice	*OsLBD12-1*	SAM size control	*AGO10*	[53]
	*Eucalyptus grandis*	*EgLBD29*	Secondary xylem formation, response to Gibberellin (GA) and indol-3-acetic acid (IAA)	-	[35]
	*Eucalyptus grandis*	*EgLBD37*	Secondary phloem formation, response to GA and IAA	-	[35]
	Citrus	*CsLOB1*	Citrus bacterial canker disease susceptibility	-	[20]
Class IE	*Arabidopsis*	*At* *LBD27*	Pollen development	-	[67,68,69]
Class II	*Arabidopsis*	*At* *LBD40*	GA response	-	[22]
	*Arabidopsis*	*AtLBD41*/ *ASL38*	Leaf adaxial-abaxial polarity	-	[88]
	*Arabidopsis*	*At* *LBD37*	Anthocyanin synthesis, nitrogen responses	*PAP1 and PAP2*	[24]
	*Arabidopsis*	*At* *LBD38*			
	*Arabidopsis*	*At* *LBD39*			
	Rice	*OsLBD37*	Nitrogen metabolism, heading date	*Ehd1*	[25,26]
	Rice	*OsLBD38*	Heading date	*Ehd1*	[26]
	*Medicago*	*MtLBD1*	Lateral roots emergence, auxin signaling	*HB1*	[58]

## Supplementary Materials

Supplementary materials can be found at https://www.mdpi.com/1422-0067/21/7/2278/s1. Additional file 1. LBD proteins of four other plants; Additional file 2. LBD proteins of six monocots; Additional file 3. LBD proteins of eight eudicots; Additional file 4. LBD protein sequences
